# Scopolamine-induced “cholinergic stress test” in the elderly

**DOI:** 10.3389/fphar.2014.00182

**Published:** 2014-08-13

**Authors:** Gustavo C. Román, Robert E. Jackson, E. Mariana Longoria, Ronald E. Fisher

**Affiliations:** ^1^Department of Neurology, Methodist Neurological Institute, Houston Methodist HospitalHouston, TX, USA; ^2^Department of Neurology, Professor of Neurology, Weill Cornell Medical College of Cornell UniversityNew York, NY, USA; ^3^Department of Medicine, Houston Methodist HospitalHouston, TX, USA; ^4^Department of Medicine, Clinical Professor of Internal Medicine, Weill Cornell Medical College of Cornell UniversityNew York, NY, USA; ^5^Division of Cognitive Aging and Geriatric Psychiatry, Mexican National Institute of Neurology and NeurosurgeryMéxico DF, México; ^6^Departments of Radiology and Neuroscience, Assistant Professor in Radiology and Neuroscience, Baylor College of MedicineHouston, TX, USA; ^7^Director of Nuclear Medicine, Houston Methodist HospitalHouston, TX, USA

**Keywords:** scopolamine, delirium, dementia, amnestic, cognitive disorders, anticholinergic, mild cognitive impairment, seasickness prevention

## Introduction

The excellent review article published in *Frontiers in Pharmacology* by Antor et al. (9 April 2014), concludes: “Clinical trials with transdermal scopolamine have consistently demonstrated its safety and efficacy in postoperative nausea and vomiting (PONV). Thus, scopolamine is a promising candidate for the management of PONV in adults as a first line monotherapy or in combination with other drugs. In addition, transdermal scopolamine might be helpful in preventing postoperative discharge nausea and vomiting owing to its long-lasting clinical effects.”

We believe a cautionary note is required before granting unrestricted endorsement to this treatment. In our experience, the use of scopolamine in the elderly may trigger episodes of amnestic delirium. The following case exemplifies the effect of transdermal scopolamine for prevention of motion sickness during a vacation ship cruise by a previously normal elderly woman.

## Case report

A healthy, young-looking, intelligent and cheerful 80-year-old woman was evaluated at the Houston Methodist Hospital Memory Clinic because of an episode of “scopolamine intoxication” that occurred on November 12, 2010. She flew from Texas to Hawaii and prior to boarding a cruise ship applied a post-auricular transdermal scopolamine patch to prevent seasickness. Her last memories of the vacation include arriving to Hawaii and boarding the cruise ship. She has no recollection after becoming confused, delirious, agitated, and experiencing visual hallucinations. Because of her markedly altered mental status she was disembarked, admitted to a local hospital in Hawaii and eventually flown back to Texas 5 days later.

**Figure 1:**
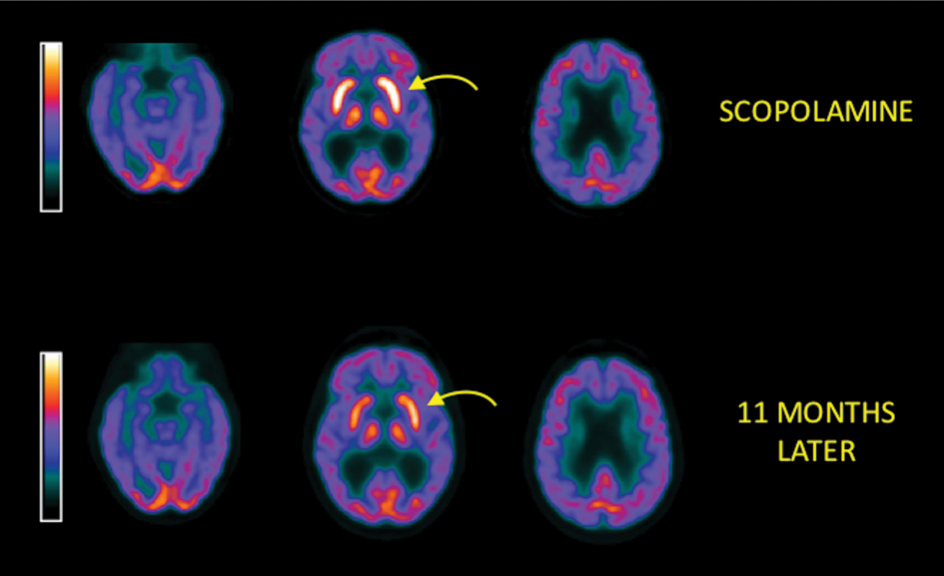
**Top**: Axial brain slices of a 2-deoxy-2-(^18^F)fluoro-D-glucose positron emission tomography (FDG-PET) scan dated 11/30/10: left slice = level of temporal lobes, middle = basal ganglia, right = frontal and parietal cortex. Scan shows markedly increased tracer uptake in the basal ganglia (arrow indicates left striatum), which appears striking against the normal uptake diffusely in the cortex. Basal ganglia to cortex ratio = 2.1. This finding has not been described consistently for any neurological disorder and is presumably secondary to scopolamine. Color bar indicates standardized uptake value (SUV) and runs from 0 (black) to 14 (white). On this display scale, the basal ganglia are actually “burned out” bright, with an SUV of 20. **Bottom:** FDG-PET performed on 10/25/11 demonstrating essentially normal tracer uptake in the basal ganglia (arrow) and cortex. The basal ganglia to cortex ratio = 1.5. This represents a dramatic improvement from the previous scan. The striking interval improvement in the scan supports the hypothesis that the original PET abnormality was a medication effect. Color bar indicates SUV and runs from 0 (black) to 14 (white).

On November 30, 2010 her neurological and neuropsychological evaluation was consistent with Mild Cognitive Impairment - Amnestic type. A 2-deoxy-2-(^18^F)fluoro-D-glucose (FDG) positron emission tomography (PET) brain scan showed markedly increased tracer uptake in the basal ganglia bilaterally (Figure 1, top). The standardized uptake value (SUV) in basal ganglia was 20, with a basal ganglia to parietal cortex ratio of 2.1. Since her clinical course was so acute with rapid resolution over a 6- to 8-week period it was felt that she did not have dementia. She completely recovered from this episode and a PET scan was repeated on October 25, 2011 showing a near-normal tracer distribution in the basal ganglia and cortex. The SUV of the basal ganglia was now 12, which is normal, and the basal ganglia to cortex ratio was now 1.5, also normal (Figure 1, bottom).

## Discussion

In our experience, scopolamine-induced amnestic delirium in the elderly is not exceptional. Seo et al. (2009) reported 7 women with similar presentations seen at their Memory Clinic in a period of 6 years with a mean age of 72.4 years (range, 65–82 years). These authors found seven additional cases reported in the literature ranging in age from 60 to 84 years. We believe these untoward scopolamine effects are not to be found in the young-age patients in surgical series of elective cesarean sections, or in outpatient laparoscopic, plastic surgery, or breast augmentation surgery compiled by Antor et al. (2014). This may be due to the aging brain's particular susceptibility to central nervous system (CNS) drugs. While anticholinergic drug use in older adults has been cautioned for many years, scopolamine specifically only made it to the Beers Criteria in 2012 (The American Geriatrics Society, 2012).

The transdermal scopolamine patch applied to the post-auricular area contains 1.5 mg of scopolamine; the system releases *in-vivo* approximately 1.0 mg of scopolamine over a 72-h period with an average plasma concentration of 87 pg/mL of free scopolamine; detectable levels are found within 4 h with a peak level at 24 h (reviewed by Antor et al., 2014). Scopolamine is a high-affinity selective competitive antagonist of G protein-coupled muscarinic receptor for acetylcholine. Scopolamine penetrates the blood-brain barrier, readily blocking cholinergic transmission in the CNS. It not only affects the vestibular nuclei and the vomiting center, but also the extensive cholinergic network that originates in the Nucleus Basalis of Meynert.

We hypothesize that the CNS cholinergic blocking effect of scopolamine induces a “cholinergic stress test” resulting in amnestic delirium in elderly patients who have low central cholinergic reserves as a result of age, subclinical Alzheimer's disease, or vascular lesions affecting the cholinergic pathways in the periventricular white matter. It is well demonstrated that the elderly are more sensitive to the cognitive effects of scopolamine than young individuals, probably due to central cholinergic system deficits associated with aging (Flicker et al., 1992; Naranjo et al., 2000). Age is also relevant in the reversible reduction of cerebral blood flow and glucose consumption produced by cholinergic blockage induced by scopolamine (Blin et al., 1997).

The effects of acute muscarinic blockade on brain glucose metabolism have not been well studied, but we found a striking and reversible hypermetabolism of the basal ganglia, a very unusual finding on PET imaging. The mechanism of the effect is far from understood, but muscarinic cholinergic synapses exert powerful and complex effects on neurotransmission in the basal ganglia, with muscarinic receptors mediating a wide variety of excitatory and inhibitory effects in both the direct and indirect pathways (reviewed by Benarroch, 2012). Muscarinic synapses are also widely present in the cortex and likely contribute importantly to the scopolamine clinical effect we observed, but this neurochemical change did not produce a detectable effect on glucose metabolism in the cortex.

In summary, we believe that the transdermal scopolamine patch for prevention of PONV or motion sickness should be used with caution in most elderly patients. This group includes those with a history of glaucoma or urinary problems. Scopolamine should be avoided completely in subjects with a diagnosis of Mild Cognitive Impairment or when the patient's relatives acknowledge the occurrence of age-related memory loss.

### Conflict of interest statement

The authors declare that the research was conducted in the absence of any commercial or financial relationships that could be construed as a potential conflict of interest.
